# Phytochemical Profiling, Acute Toxicity, and Hepatoprotective Effects of *Anchusa Limbata* in Thioacetamide‐Induced Liver Cirrhosis in Rats

**DOI:** 10.1002/fsn3.4544

**Published:** 2024-11-21

**Authors:** Khaled Abdul‐Aziz Ahmed, Ahmed A. J. Jabbar, Mohammed M. Hussein M. Raouf, Ayman M. Al‐Qaaneh, Rawaz Rizgar Hassan, Musher Ismael Salih, Ramzi A. Mothana, Gadah Abdulaziz Al‐Hamoud, Mahmood Ameen Abdulla, Sidgi Hasson, Parween Abdul‐samad Ismail

**Affiliations:** ^1^ Department of Medical Laboratory Sciences, Faculty of Allied Medical Sciences Al‐Ahliyya Amman University Amman Jordan; ^2^ Department of Medical Laboratory Technology, Erbil Technical Health and Medical College Erbil Polytechnic University Erbil Iraq; ^3^ Department of Biomedical Sciences, College of Applied Science Cihan University‐Erbil Erbil Iraq; ^4^ Department of Allied Health Sciences Al‐Balqa Applied University (BAU) Al‐Salt Jordan; ^5^ Department of Pharmaceutical Technology Faculty of Pharmacy, Jordan University of Science and Technology (JUST) Irbid Jordan; ^6^ Department of Medical microbiology, College of Science Knowledge University Erbil Iraq; ^7^ Department of Chemistry, Faculty of Science and Health Koya University Koya Iraq; ^8^ Department of Pharmacognosy, College of Pharmacy King Saud University Riyadh Saudi Arabia; ^9^ Department of Medical Analysis, Faculty of Applied Science Tishk International University Erbil Iraq; ^10^ School of Pharmacy and Biomolecular Sciences Liverpool John Moores University Liverpool UK; ^11^ Chemistry Department, College of Education Salahaddin University Erbil Iraq

**Keywords:** antioxidant enzymes, histology, immunohistochemistry, liver cirrhosis

## Abstract

Evaluation of *Anchusa species* of the family Boraginaceae during previous investigations determined numerous therapeutic potentials against inflammatory‐related diseases. The present study evaluates the phytochemical, acute toxicity, and hepatoprotective effects of methanolic extracts of *Anchusa limbata* (MEAL) against thioacetamide (TAA)‐induced liver injury in rats. The phytochemical profiling of MEAL followed a Folin–Ciocalteu and 10% AlCl3 procedure using a spectrophotometer. Thirty rats were divided into 5 groups: Normal (A) and TAA control rats (B) treated orally with daily 10% tween 20; reference rats (C) received daily oral dose of 50 mg/kg silymarin; (D and E) rats received daily doses of 250 and 500 mg/kg MEAL, respectively. In addition, group B‐E received 3 injections of 200 mg/kg TAA weekly for 60 days. The phytochemical profiling showed increased polyphenolic (129.2 mg gallic acid equivalent/g) and flavonoid (105.3 mg quercetin equivalent/g extract) contents in MEAL. The TAA intraperitoneal injection caused significant hepatic dysfunctionality (lowered total protein, 54.7 g/L; albumin levels, 7.8 g/L), hepatotoxicity, and necrotized cell proliferation. TAA hepatotoxicity resulted in an increased expression of proliferating cell nuclear antigen (PCNA), TGF‐β1 tissue expression, liver enzymatic leakage, and oxidative stress biomarkers, while it reduced pro‐apoptotic Bcl‐2–associated X protein (Bax) proteins and inflammatory mediators (TNF‐α and IL‐6) and increased IL‐10. Conversely, MEAL treatment ameliorated the TAA‐induced hepatotoxicity and restored liver functions. The present hepatoprotectives of MEAL could be attributed to its increased polyphenolic and flavonoid contents, which require further isolation and identification of molecules underlying such therapeutic actions.

## Graphical abstract

The phytochemical profiling showed increased polyphenolic (129.2 mg GAE/g) and flavonoid (105.3 mg EQ/g extract) contents in methanolic extract of *Anchusa limbata*. The present of MEAL supplementation ameliorated TAA‐mediated hepatoxicity in rats by modulating several molecular mechanisms. MEAL oral ingestion reduced expression of proliferating cell nuclear antigen (PCNA), TGF‐β1 tissue expression, liver enzymatic leakage, and oxidative stress biomarkers, while it up‐regulated pro‐apoptotic Bcl‐2‐associated X protein (Bax) proteins and anti‐inflammatory mediators (IL‐10).

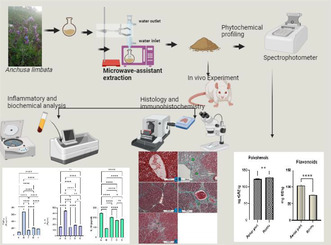

## 1 Introduction

Liver cirrhosis is a major death risk factor in patients with chronic liver disease according to statistics that nearly 2 million deaths occur each year, which accounts for 4% of all global deaths (one in every 25 deaths worldwide); almost two‐thirds of all liver‐linked deaths occurred in men (Devarbhavi et al. 2023). Moreover, World Health Organization data from 2018 declared an increased age‐standardized rate of liver cirrhosis with values of 15+ for every 100,000 individuals (Choi et al. 2023). Liver injury and hepatic cirrhosis develop as a result of different exogenous and endogenous factors, including toxin exposure, viral infection, alcoholism, metabolic disorder, diabetes, biliary stones, and non‐alcoholic fatty liver (Ko, Yoon, and Jun 2023). The prognosis and the severity of liver disease can be different in each individual because of cause variability, area of damaged hepatic tissue, pro‐fibrogenic paths, and pro‐fibrogenic myofibroblast factors (El‐Baz, Salama, and Salama 2019). Moreover, liver disease characterized by increased accumulation of extracellular matrix proteins (collagen) commonly found in patients with chronic liver diseases. The immune and inflammatory responses to chronic inductions are considered a key factor in the initiation and progression of Cirrhosis. At first, hepatic cirrhosis starts with liver injury induced by numerous etiological factors, initiating inflammatory infiltration, and ignition of the inflammatory cascade (Lim et al. 2018). Moreover, perisinusoidal cells (hepatic stellate cells) are converted into fibroblast‐like cells capable of generating several types of collagen proteins along with laminin. Consequently, these liver protein alterations can have significant modulatory actions on the structure and function of hepatocytes subsequently affecting overall liver performance (Feng et al. 2024). Transforming growth factor‐β (TGF‐β) is a profibrogenic cytokine that modulates different stages of chronic liver disease by inflammation and fibrosis progressing into cirrhosis and liver carcinoma. During chemically induced liver damage, increased production of TGF‐β cytokine stimulates hepatic stellate cells and fibroblasts that enhances wound healing action (myofibroblast formation and deposition of extracellular matrix) (Zhang et al. 2019).

Oxidative stress is a well‐known key player in the progression of liver injury in TAA‐mediated hepatotoxicity that ultimately leads to liver cirrhosis according to numerous research reports (Abduljabbar and Mehmetcik 2019; Jabbar et al. 2023a, 2023b). The TAA liver injury occurs once there is an increased amount of cytochrome P450 oxidases that converts TAA into less toxic substance, consequently generating increasing amount of reactive oxygen species, lipid peroxidation (MDA), and release of pro‐inflammatory chemicals (tumor necrotic factor‐α, interleukin‐6). Reactive oxygen molecules can damage cellular proteins, membrane organelles, and genetic materials. Once they reach liver, TAA is converted by CYP450 2E1 enzyme into a number of oxidant molecules, TAA‐S‐oxide and TAA‐S‐dioxide; the TAA‐S‐dioxide molecule can provoke oxidative stress through peroxidation of the lipid bilayers in the hepatocyte membrane. A single dose of TAA (50–300 mg/kg) can initiate liver necrosis by interacting with liver macromolecules in the pericentral area; for example, downregulating the pro‐apoptotic Bax protein (a Bcl‐2‐like protein enhances the disruption of membrane permeability, thereby enhancing cytochrome c release in damaged hepatocytes) (Abdul‐Aziz Ahmed et al. 2024; Jabbar and Alamri 2024). Chronic liver disease found alongside fibrosis, liver injury, and liver dysfunction increases non‐functional plasm enzymes, including aspartate aminotransferase (AST), alkaline phosphatase (ALP), and Alanine aminotransferase (ALT) (Niederreiter and Tilg 2018). PCNA is a nuclear protein that is considered a good indicator of cell proliferation. PCNA is considered a strong biomarker for the prognosis and survival rate in most solid cancer types, hepatocellular carcinoma. PCNA is an accurate reflector for the rates of necrotized cellular proliferation and transcriptional process (DNA formation) because of its aggregation in the late G1 and early S stages of mitosis. Early detection of PCNA intensity in hepatic biopsy of liver cirrhotic patients revealed increased expression of these proteins, indicating uncontrolled cellular proliferation (Shivaramu et al. 2024).

Liver cirrhosis patients exhibit increased deposition of extracellular matrix (ECM) collage because of increased production of transforming growth factor beta 1 (TGFβ1), a pro‐inflammatory cytokine that provokes the initiation of liver cirrhosis via activation of hepatic stellate cells (HSCs) and increases α‐SMA formation in the ECM. Silymarin is a bioactive extract of milk thistle that has been confirmed as a strong antioxidant and hepatoprotective agent. As a flavonolignan mixture, silymarine has been used widely by global researchers as a reference drug to alleviate TAA‐induced hepatotoxicity in rats (Gillessen and Schmidt 2020; Xiang et al. 2020).

*Anchusa* species is a well‐known flowering plant that grows in the Mediterranean region in sandy soil overlying saline flats and on the banks of rivers (Ghazanfar and McDaniel 2015), in which 9 species have been found in Iraq, especially in Diyala, Baquba, Liwa, and in Kurdistan, Haji Omaran district and the edge of shallow pools. These plants include *A. aegyptiaca*, *A. arvensis*, *A. italica*, *A. strigosa*, *A. hispida*, *A. italifolia*, *A. aucheri*, *A. officinalis*, *A. limbata* (Townsend and Guest 1985; احمد 2008). Ethnobotanical records in Iraqi Kurdistan declared different traditional uses of *Anchusa* species for various health issues including inflammation, pain, and wounds (Pieroni, Ahmed, and Zahir 2017; Pieroni et al. 2019; Galalaey et al. 2021). Flowers and leaves of the *Anchusa* plant have been boiled or fried in oils with onion and eggs before ingestion to alleviate abdominal pain and digestive problems (Pieroni, Ahmed, and Zahir 2017). Phytochemical profiling of *Anchusa* species revealed polyphenolic, flavonoids, and terpenoids as major chemical constituents responsible for various biological potentials including anti‐inflammatory, antioxidant, and immunomodulatory actions (Liu et al. 2020; Paun et al. 2020; Wang et al. 2020).

To our knowledge, no previous records are available on the protective effects of *Anchusa limbata* against hepatotoxicity. Accordingly, our investigation conducted to explore the hepatoprotective action of methanolic extracts of aerial parts of *Anchusa limbata* in TAA‐induced hepatotoxic rats with the phytochemical profiling of MEAL using conventional techniques and subsequently determine the underlying pathophysiology and pharmacological pathways.

## 2 Materials and Methods

### 2.1 Plant Collection and Extract

**FIGURE 1:**
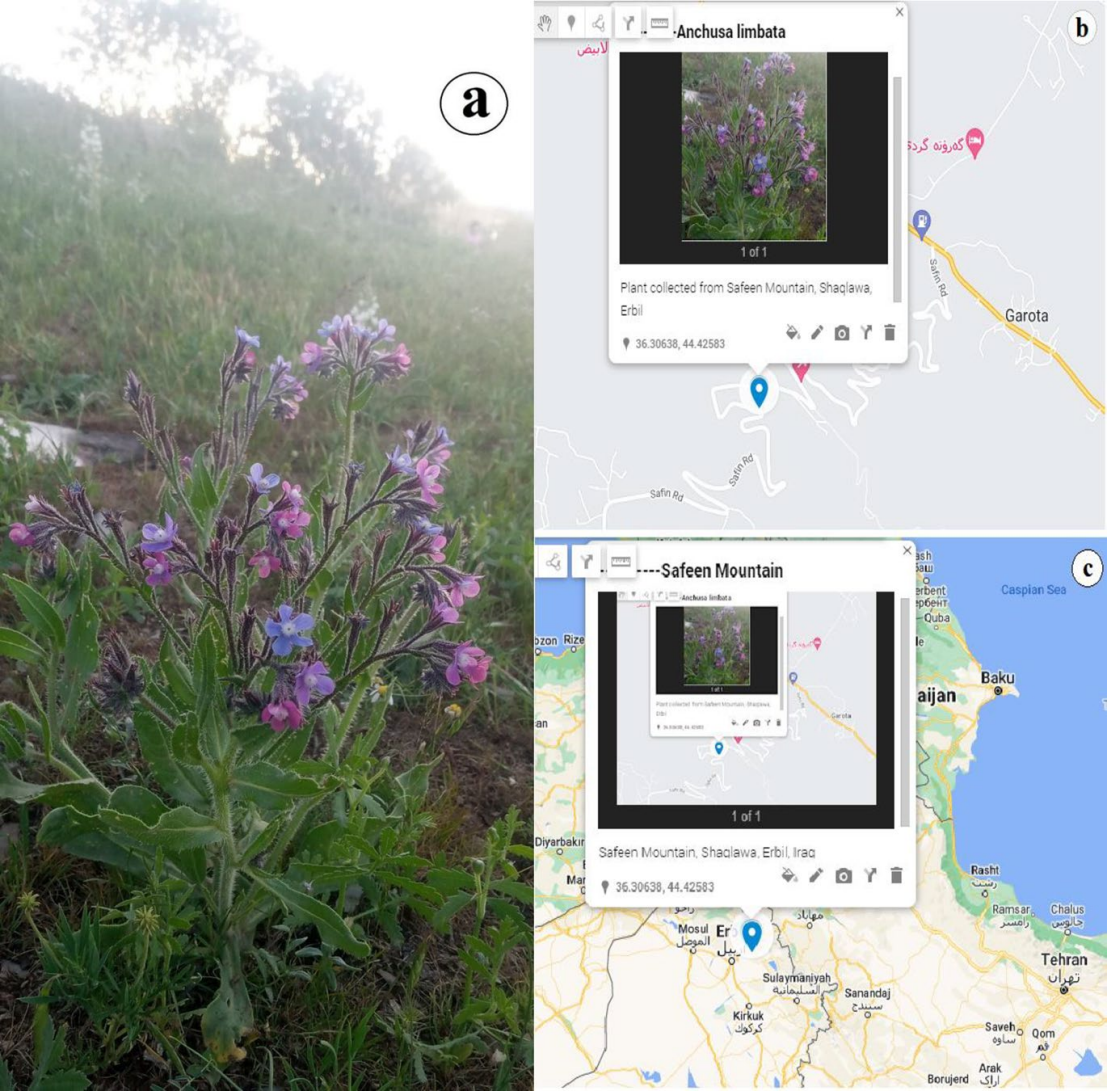
*Anchusa limbata* (a) collected from Safeen Mountain (b) located in Erbil, Iraq (c).

The *Anchusa limbata* aerial parts were collected from Safeen Mountain, Iraqi Kurdistan during spring 2022 (Figure 1). After authentication by Prof. Dr. Abdullah Sh. Sardar, Voucher number obtained (7310) from Herbarium of educational college, Salaheddin University. After washing and drying in a shadow place, plant parts were grinded into a coarse powder and mixed with a methanol: water (95:5 ratio) solution. The mixture was moved into an ultrasonic bath for 2 h as an incubation technique at 37°C. Rotary evaporation and rotary vacuum evaporation were implemented to remove excess solvents and then, freeze‐dried. The dried extract (yield 4.3%) was kept in dark glass bottles with a vacuum desiccator (Esmeeta et al. 2022).

### 2.2 Phytochemical Investigation

#### 2.2.1 Detection of Polyphenolic Content

The present profiling of the polyphenolic content in the aerial parts and roots of *A. limbata* followed–Ciocalteu procedure with slide changes by applying gallic acid as a reference (Jabbar 2021). Briefly, 0.2 mL of Folin Ciocalteu reagent was mixed with 7.5% Na_2_CO_3_ and 0.2 mL of MEAL. After 30 min, the mixed solution was transferred into a cuvette for the absorbency determination by spectrophotometry (760 nm), and the concentration of polyphenolic was calculated by using a calibration equation. That is, *y* = 0.0001*x* + 0.047 and *R* ^2^ = 0.8348.

#### 2.2.2 Detection of Flavonoid Content

The flavonoid contents of the aerial parts and roots of *A. limbata* were determined using the below procedure. The plant extract (MEAL) was added to a container with 2 mL of 10% AlCl3 and 7.2 mL of ethanol. After 40 min, the mixture was transferred into a cuvette for spectrophotometric evaluation (430 nm), and the flavonoid concentration was determined by the equation obtained from the calibration range using quercetin as a reference ingredient. That is, *y* = 0.0019*x* + 0.0558 and *R* ^2^ = 0.9844.

### 2.3 Ethical Approval

The laboratory handling of animal rats follows the international regulations for animal care set by National scientific instructions (MacArthur Clark and Sun 2020). The study protocol was approved by the Tishk International University (No. 12, 19/11/2023, M.A.A.).

### 2.4 Chemicals

The hepatotoxic inducer (Thioacetamide, TAA) and standard silymarin drug were obtained from Sigma‐Aldrich (Merk, Germany). TAA was dissolved in a flask containing 10% Tween 20, liquefied, and used as a stock solution. TAA solution was given by intraperitoneal injection (3 doses weekly) to rats for 2 months. The reference drug (silymarin) was prepared in 10% tween 20, which was given in 50 mg/kg to rats (Abood et al. 2020). Ketamine and Xylazine anesthesia were purchased from Bayer Company, Istanbul, Turkey.

#### 2.4.1 Acute Toxicity Experiment

Sprague Dawley male rats (7–8 weeks) weighing about 170–180 g were obtained from the Faculty of Science, Cihan University‐Iraq. Thirty rats were distributed into three wide‐mesh wire cages (avoiding coprophagia), and they had a standard diet and tap water for 7 days of adaptation (Ahmed et al. 2024b). After that, the three rat clusters underwent overnight fasting and they were treated as follows the next day:

$$\begin{array}{c} \text{Normal rats treated with}\;10\%\text{Tween}\;20\;\left(5\;\mathrm{mL}/\mathrm{kg}\right):\\ B,\text{rats supplemented with}\;2\;g/\mathrm{kg}\;\text{of MEAL};\\ C,\text{rats ingested with}\;5\;g/\mathrm{kg}\;\text{of MEAL}. \end{array}$$

Rats were not allowed to have food following 3–4 h after supplementation. The observation started immediately and continued for the next 48 h for any potential toxic incidence or abnormalities. After 14 days, all rats received an overdose of anesthesia (ketamine and xylazine) and were sacrificed. The intracardial blood samples were analyzed for biochemical contents, and various organs were examined by histological assays using hematoxylin and Eosin stain (Wahab et al. 2023).

#### 2.4.2 Hepatoprotective Trial

##### 2.4.2.1 Experimental Design

Thirty mature male rats were obtained from the Faculty of Science, Cihan University‐Iraq. The animals were housed in plastic cages (7 animals each) at a temperature of 25°C and 70% humidity, with 12 h light/ dark cycle, and had free access to standard rodent diet and water.

**FIGURE 2:**
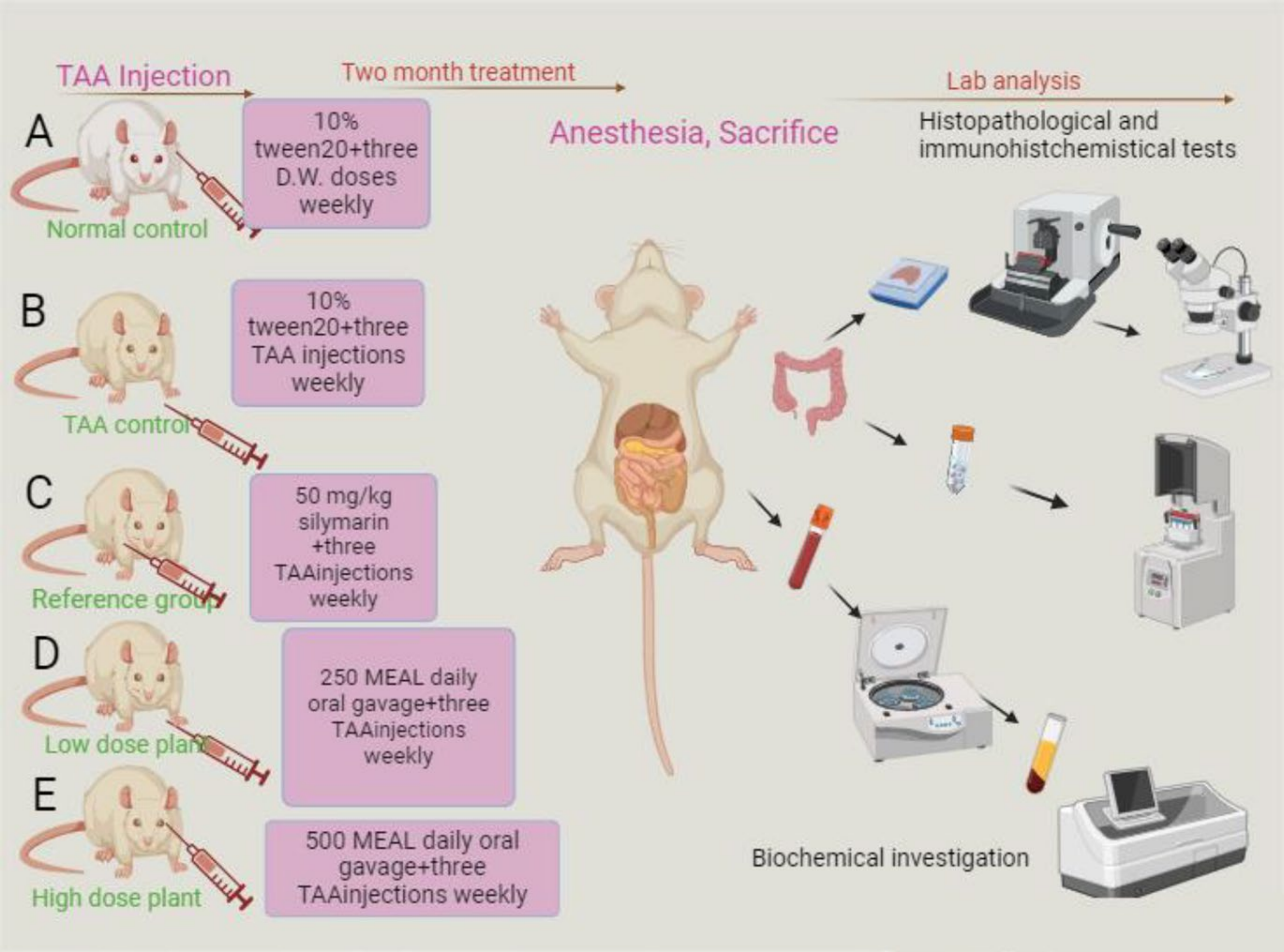
Experimental design of different treated rats included in the hepatoprotective evaluation of MEAL.

The rats were clustered into 5 wire‐mesh cages (6 rats in each group) and were given the following treatments (Figure 2):

(A) Normal control rats were administered a daily dosage of 10% Tween 20 (5 mL/kg) by oral gavage and received three intraperitoneal injections (5 mL/kg) of sterile distilled water each week for 2 months.

(B) Cirrhosis rats were administered a daily dosage of 10% Tween 20 (5 mL/kg) by oral gavage and injected with 3 intraperitoneal injections of 200 mg/kg of TAA per week for 2 months.

(C) Reference rats were administered a daily dosage of 50 mg/kg silymarin by oral gavage and injected with 3 intraperitoneal injections of 200 mg/kg of TAA per week for 2 months.

(D and E) Rats were administered daily dosages of 250 and 500 mg/kg of MEAL by oral gavage and injected with 3 intraperitoneal injections of 200 mg/kg of TAA per week for 2 months.

After that, food was removed from rats overnight and they were given 100 m/kg anesthesia (Ketamine and Xylazine) and sacrificed. The blood samples from the intracardial puncture were tested for different biochemical compounds, and the dissected livers were evaluated by histopathologic techniques (Jabbar et al. 2023c).

##### 2.4.2.2 Macroscopic Viewing of the Liver

The dissected liver organs were cleaned with normal saline (cold) and dried by the filter papers for the estimation of gross weight and gross morphological studies (Salama et al. 2018).

Liver index%=(Liver weight)/(body weight)×100

##### 2.4.2.3 Histology of Liver

The liver lobes were kept in the flask containing 10% phosphate buffered formalin for 1 day as a tissue fixation technique. After that, the liver tissue was paraffinized in paraffin blocks through a tissue processing machine (Leica, Germany). Small slices (5 μm) of the liver tissue were covered and slipped on slides and histopathological investigation included staining with hematoxylin, eosin, and Masson Trichrome stains (Lv et al. 2021; Arshad et al. 2024).

##### 2.4.2.4 Immunohistochemistry

The rat monoclonal anti‐PCNA antibody [PC10] and monoclonal anti‐actin (anti‐α‐Sm‐1) were purchased for proliferating cell nuclear antigen (PCNA); Bcl‐2–associated X protein, and Bax detections, respectively. Briefly, by utilizing poly‐L‐lysine‐coated slides, liver sections were kept in an oven (DON‐HE Series, Infitek, Shandong, China) for 25 min at 60°C. After heating, xylene was used for deparaffinization (2 × 3 min) and alcohol (series‐graded alcohol, 95%, 75%) was also used for hydration followed by running water. The antigen recovery technique was implemented using via 10 mM sodium citrate buffer after boiling in a microwave for 10 min. The slides were colored following the instructions of the producer's company (Sigma Aldrich, Merck, Germany) using tris buffered saline with 0.05% tween‐20. Briefly, 0.03% hydrogen peroxide sodium azide was utilized for blocking the endogenous peroxidase for 5 min. Liver sections were buffer washed with distal water for 3 min, cleaned, and incubated for 15 min with antibodies (PCNA and Bax) (1:200) and TGF‐β (Clone sc‐146) (1:50, diluted anti‐body) according to company's protocols. After careful washing for 3 min and kept in a humid chamber, then, it was re‐intubated for 15 min with streptavidin HRP and washed two times with distal water. The liver sections were incubated for 7 min with diaminobenzidine substrate chromogen and buffer washed again for coloring with counterstain hematoxylin (5 s). Finally, tissue sections dipped in 0.037 mol/L ammonia (5 times), cleaned with deionized water (2–5 min), and cover‐slipped for the microscopic detection of cytoplasmic brown granules and brown‐colored nuclei for the positive appearance of PCNA and Bax antigens, respectively, while TGF‐β appeared as a brown color area under the microscope. The staining intensity of PCN was found by estimating the percentage of colored cells divided by 1000 hepatocytes (Ahmed et al. 2024a).

##### 2.4.2.5 Liver Antioxidant

The liver samples (1 g) from both left liver lobes were transferred into a flask of 10 mL containing PBS solution (10%, pH 7.2) for buffering and then transferred into a homogenizer (operated at 5000 rpm, 15 min at −4°C). The supernatant was transferred into a freezer (−80°C) for later use. Different antioxidant kits of CAT, SOD, GPx, and MDA were bought from Sigma Aldrich (Merck, Germany) (Jabbar et al. 2023a).

##### 2.4.2.6 Serum Inflammatory Cytokines

The present estimation of TNF‐α, IL‐6, and IL‐10 chemicals in serum specimens obtained from experimental rats followed the instructions provided in the ELISA kit (Cusabio Biotech Co. China) (Almaimani et al. 2024).

#### 2.4.3 Biochemistry of Liver Functions

The serum samples were analyzed for the liver enzymes (ALT, AST, and ALP), liver synthetic functions (total protein and albumin), and the excretory (bilirubin) functions of the liver (Ali Abed Wahab et al. 2023).

#### 2.4.4 Statistical Analysis

The values are available as mean ± SEM. The statistical procedure was possible by SPSS, one‐way ANOVA, post hoc, and a significance level setup at *p* < 0.05. The graphs created by GraphPad Prism 9 software.

## 3 Results

### 3.1 Phytochemical Contents

**FIGURE 3:**
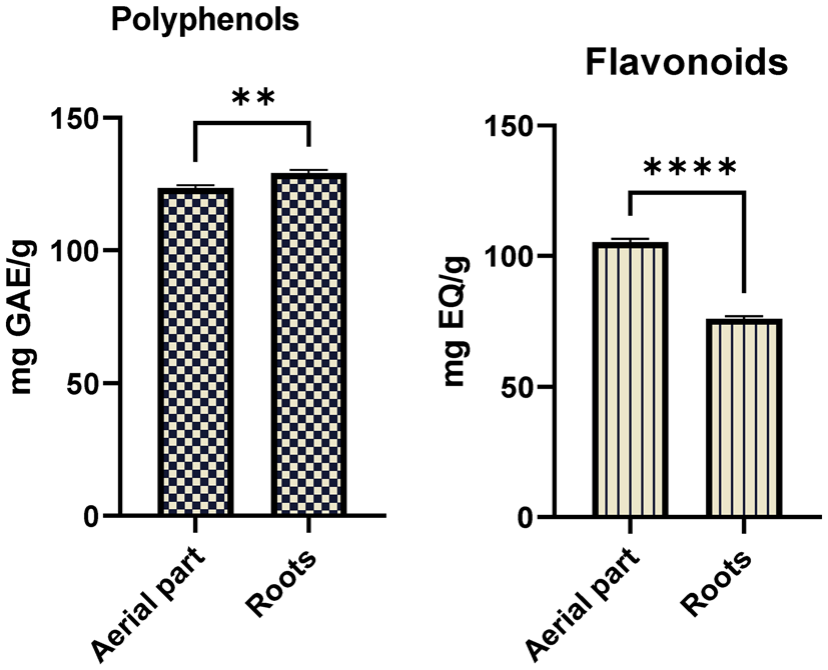
Phytochemical profile of different parts of *Anchusa limbata*. **, *p* < 0.01; ****, *p* < 0.0001.

Polyphenol concentration was calculated from a calibration curve of gallic acid (regression line). The results revealed significant (*p <* 0.01) difference between root and aerial parts of *Anchusa limbata*. The polyphenolic estimation was 123.6 and 129.2 mg GAE/g extract for aerial parts and roots, respectively (Figure 3).

The present evaluation for the flavonoid content was significantly varied (*p <* 0.0001) between roots and aerial parts of *Anchusa limabata*. The data analysis indicated noticeably higher (105.3 mgEQ/g extract) flavonoid levels for aerial parts compared to that (75.9 mgEQ/g extract) of roots (Figure 3).

### 3.2 Acute Toxicity

**FIGURE 4:**
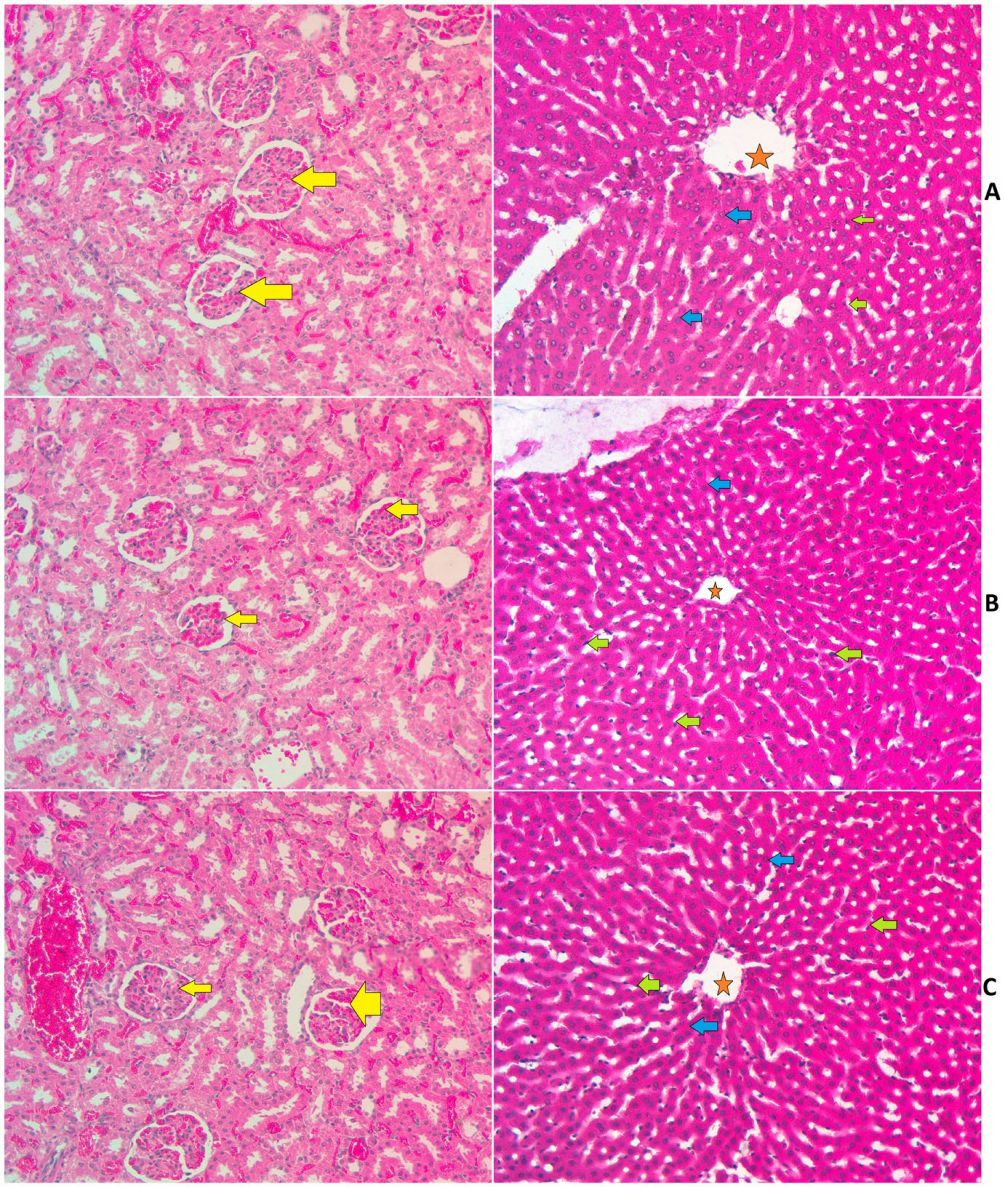
Microscopic presentation of the liver and kidney tissues of rats in acute toxicity trial. (A) normal controls fed on 10% tween 20; (B) rats ingested 2 g/kg MEAL; (C) rats ingested 5 g/kg MEAL. The alignment of the kidney and liver histological layers were very comparable between normal control and MEAL‐treated rats. The kidney tissue appeared as a normal bowman's capsule with glomeruli (yellow arrow) and adequate interlobular blood vessels as well as distal convoluted tubule and proximal convoluted tubules. The hepatic tissues appeared with a central vein (red asterisk); kupffer cell (green arrow), and normal liver cell with circular nucleus (blue arrow) for all tested rats (hematoxylin and eosin stain, 20×).

The present plant supplementation (MEAL) at 2 and 5 g/kg for 2‐week period did result in any significant physiological change or mortality in Sprague–Dawley rats. The continued regular check‐up on MEAL‐treated rats did find any noticeable toxicological changes (skin and eye color, diarrhea, tremors, salivation, irritation, locomotion, or convulsion). The food and water intake were very comparable between normal control and MEAL‐treated rats. The structure tissue arrangement of liver and kidney organs were similar based on microscopic examinations of slides stained with hematoxylin and eosin. The serum biochemical components of MEAL‐treated rats were similar compared to that of normal control rats (Tables 1 and 2, Figure 4A–C). Based on the outcomes, the expected toxicity dose of MEAL would be higher than 5 g/kg. The estimated liver (Table 1) and kidney (Table 2) parameters were found non‐significant varied between supplemented rats and normal controls.

**TABLE 1:** Effect of MEAL supplementation on liver function tests.

Animals group	ALP (IU/L)	ALT (IU/L)	AST (IU/L)	T. Bilirubin (μmol/L)	T. Protein (g/L)	Albumin (g/L)
A	91.3 ± 4.4	41.3 ± 3.8	61.3 ± 3.4	1.7 ± 0.02	80.2 ± 4.2	24.5 ± 3.9
B	73.5 ± 3.8	44.7 ± 3.9	63.2 ± 3.7	1.5 ± 0.03	69.2 ± 3.4	23.3 ± 4.3
C	78.3 ± 3.8	37.4 ± 4.2	64.2 ± 3.3	1.42 ± 0.08	74.3 ± 3.4	27.4 ± 4.4

*Note:* Group A, rats received 10% tween 20; group B, rats supplemented with 2 g/kg of MEAL; group C, rats ingested with 5 g/kg of MEAL, respectively.

Abbreviations: ALP, alkaline phosphatase; ALT, alanine transferase; AST, aspartate transferase.

**TABLE 2:** Effects of MEAL administration on kidney functional parameters.

Animal groups	Sodium μmol/L	Potassium mm/L	Chloride mmol/L	Urea mmol/L	Creatinine μmol/L
A	143.2 ± 4.3	6.6 ± 1.5	105.4 ± 3.4	4.4 ± 0.5	43.7 ± 4.8
B	140.7 ± 3.4	5.6 ± 3.2	119.3 ± 3.2	5.3 ± 1.3	39.2 ± 3.9
C	143.4 ± 3.8	5.4 ± 1.4	103.4 ± 3.3	5.2 ± 1.1	42.2 ± 3.7

*Note:* A, normal cluster received 10% tween 20; B and C rats ingested 2 and 5 g/kg MEAL, respectively.

The present oral supplementation at 2 and 5 g/kg MEAL to rats revealed non‐significant alteration in the kidney functional parameters and kidney histological structure compared to values of normal control rats (Tables 1 and 2, Figure 4).

### 3.3 Hepatoprotectives of MEAL

#### 3.3.1 Body and Liver Masses

The present evaluation of body weight and liver mass found significant variations between different treated experimental rats. The normal control rats had body weight (305.2 g) within normal range. The TAA control rats showed significantly reduced body weight (174.5 g) compared to silymarin or 250 and 500 MEAL‐treated rats with values as 293.4, 225.4, 268.3 g, respectively. Furthermore, rats supplemented with 500 mg/kg MEAL restored body weight, which was very similar to silymarin‐treated rats (Table 3).

**TABLE 3:** Effect of MEAL on body parameters of rats administered TAA hepatotoxic compound.

Groups	Body weight (gm)	Liver weight (gm)	Liver Index LW/BW %
A	305.2 ± 3.6^a^	7.3 ± 0.3^a^	2.39^a^
B	174.5 ± 3.2^d^	10.6 ± 0.2^d^	6.07^c^
C	293.4 ± 3.5^b^	8.5 ± 0.3^c^	2.89^a^
D	225.4 ± 3.3^c^	9.9 ± 0.4^b^	4.38*^b^*
E	268.3 ± 5.1^b^	9.3 ± 0.3^b^	3.46^b^

*Note:* Values with same letters inside exact column means non‐significant variance at *p < 0.05*. A, normal control; B, rats had only TAA intraperitoneal injection; C, rats had TAA + Silymarin; D, rats had TAA + 250 mg/kg MEAL; E, rats had TAA + 500 mg/kg of MEAL.

The present liver weightiness of rats was statistically different as a result of different oral and intraperitoneal injections (TAA). The liver weight of normal controls rats was found as 7.3 g and liver index values as 2.39%, which were significantly less than that in the TAA, silymarin, or MEAL‐treated rats. Rats treated only with TAA control rats had significantly increased liver weight (10.6 g) and higher liver index (6.07%) than that of normal control (7.3 g, 2.38%, respectively), silymarin (8.5 g, 2.89%, respectively), or 250 mg/kg MEAL (9.9 g, 4.38%, respectively) and 500 mg/kg MEAL (9.3 g, 3.46%, respectively)‐treated rats. The present supplementation of rats with 500 mg/kg of MEAL caused significant retention of liver weightage and liver index with normal range, which was very comparable to that of silymarin‐treated rats (Table 3).

#### 3.3.2 Microscopic Results

**FIGURE 5:**
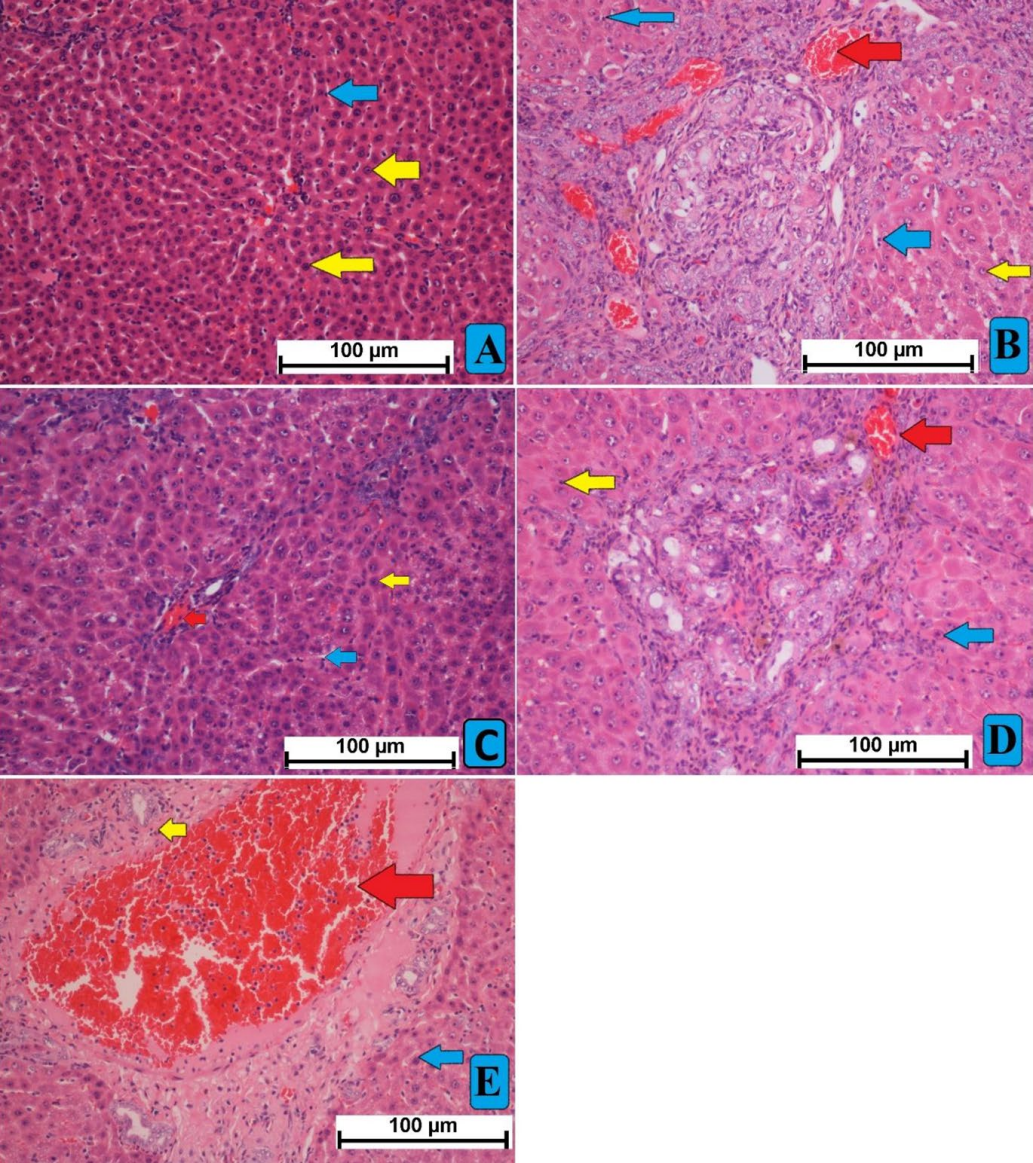
Microscopic views of liver tissus stained with H&E, hematoxylin, and eosin. (A) normal rats showed usual tissue structure arrangement; (B) TAA control rats showed increased cellular proliferation and inflammatory area. (C) rats received TAA+ 50 mg/kg silymarin had reduced hepatic injury shown by fewer micronodules and less inflammatory cells fibrous septa. (D) rats had TAA + 250 mg/kg MEAL showed moderate hepatic damage represented by reduced less inflammation and lowest cellular proliferation compared to treated groups. (E) rats that received TAA + 500 mg/kg MEAL revealed similar hepatic structural arrangement compared to that of silymarin‐treated, which were shown with fewer surface lower inflammatory infiltrations and lower tissue layer disorganization compared to TAA control rats. Nikon microscope (Y‐THS, Japan) 20× magnification. Central vein (red arrow); hepatic round cell with cytoplasm (blue arrow); and kupffer cell (yellow arrow).

The histological characterization of hepatic tissues from different treated rats revealed different levels of surface tissue alterations as a result of different treatment strategies following TAA injection (Figure 5). Normal controls (A) showed intact liver tissue with the usual criteria of cellular arrangement liver tissue without any inflammation signs or necrosis. However, the TAA control rats (B) disclosed severe liver tissue damage represented by endothelial tissue injury, ambiguous nucleus, and elevated cytoplasmic vacuoles, all of these denoting severe inflammation and tissue necrotic condition. Moreover, the parenchymal layers were seriously altered as a result of fibrous septa that adjust the collagen bond in the hepatic triangles, denoting various micro‐ and macro‐nodules in the liver cells. These nodules were found inside bundles of connective tissues that separate the liver into several lobules, accompanied by increased inflammation rate and hepatic necrosis. The silymarin‐treated rats (C) revealed noticeable protective effect against TAA‐hepatotoxicity shown by less inflammatory cell infiltration, fewer centrilobular tissue necrosis, and clearer round nucleus compared to the cirrhotic control group (B). Moreover, hepatic lobules were more organized, and capillary veins were distributed across connective tissues. The MEAL‐treated rats (D, 250 mg/kg; E, 500 mg/kg) had resisted the TAA‐induced hepatic damage shown by fewer tissue necrosis in centrilobular, fewer cirrhosis scores, less nucleus and tissue cirrhosis, and reduced vacuolization, which were significant compared to group B (cirrhosis rats) but not as significant as silymarin‐treated. Moreover, MEAL treatment led to less tissue penetration, fewer necrotic zones, and higher parenchymal cell regenerations (endothelial and sub‐endothelial layers) in TAA‐induced hepatotoxic rats (Figure 5, H&E).

**FIGURE 6:**
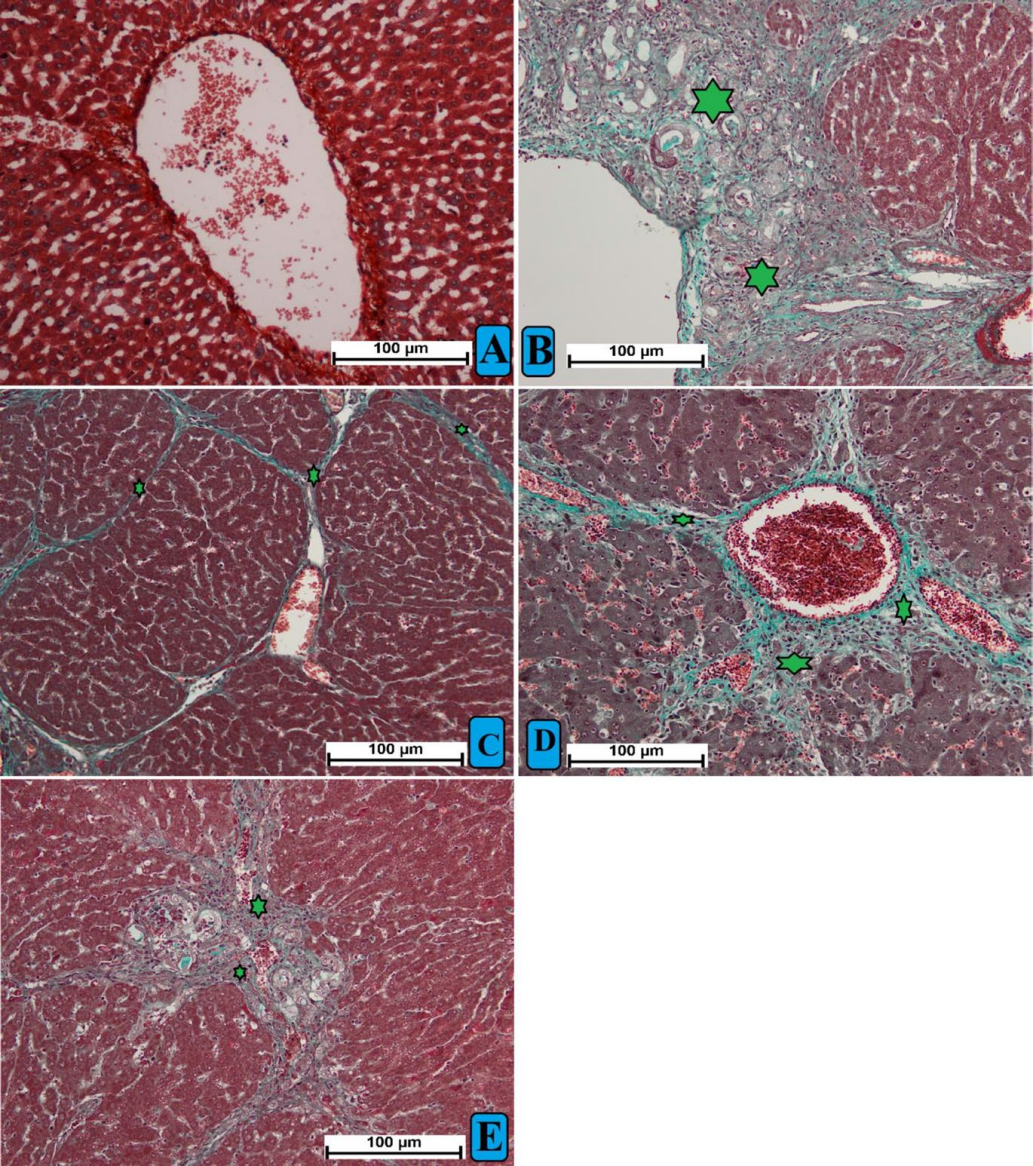
Histological views of liver tissue stained with MT, Mason trichrome. (A) normal control rats showed normal structure tissue arrangement without any sign of collagen deposition; group (B) fibrotic rats showed increased elongated bile duct, large size fibrous septum, and increased collagen deposition about central vein (green asterisk); (C) rats received TAA+ 50 mg/kg silymarin had reduced hepatic injury shown by small fibrous septa and very decreased collagen deposition; (D) rats had TAA + 250 mg/kg MEAL showed moderate hepatic damage represented by less tissue disruption, reduced inflammation, and lower collagen deposition compared to group (B); (E) rats received TAA + 500 mg/kg MEAL revealed similar hepatic structural arrangement compared to that of silymarin‐treated.

The hepatic tissues staining technique by Masson's trichrome revealed a disappearance in the tissue collagen deposition in different treated rats (Figure 6). The liver tissue sections of normal control rats (A) showed without any obvious signs of collagen depositions. The TAA control rats (B) exhibited increased collagen deposition around the central vein, indicating severe structural liver damage. Rats treated with silymarin (C) had very reduced collagen deposition, denoting the lowest hepatic cirrhosis. Rats ingested 250 mg/kg of MEAL (D) showed moderate collaged deposition and moderate congestion about the central vein. The rats ingested with 500 mg/kg of MEAL (E) had mild collagen deposition and mild congestion around hepatic central vein. MEAL or Silymarin suppressed inflammation, mononuclear cell aggregation, necrotizing hepatocytes, and fibrous proliferation in connective tissues which is induced by TAA. Accordingly, the liver tissue structure maintained their closely normal lobular arrangement. The results validate the protective effect of MEAL or silymarin in contradicting effect of TAA‐induced hepatotoxicity. A histological evaluation of liver tissue ingested with MEAL lowered grades of cirrhosis, nevertheless less necrotized tissue area, less vacuoles in cytoplasm, and nucleic alteration.

#### 3.3.3 Immunohistochemical Protein Expression

**FIGURE 7:**
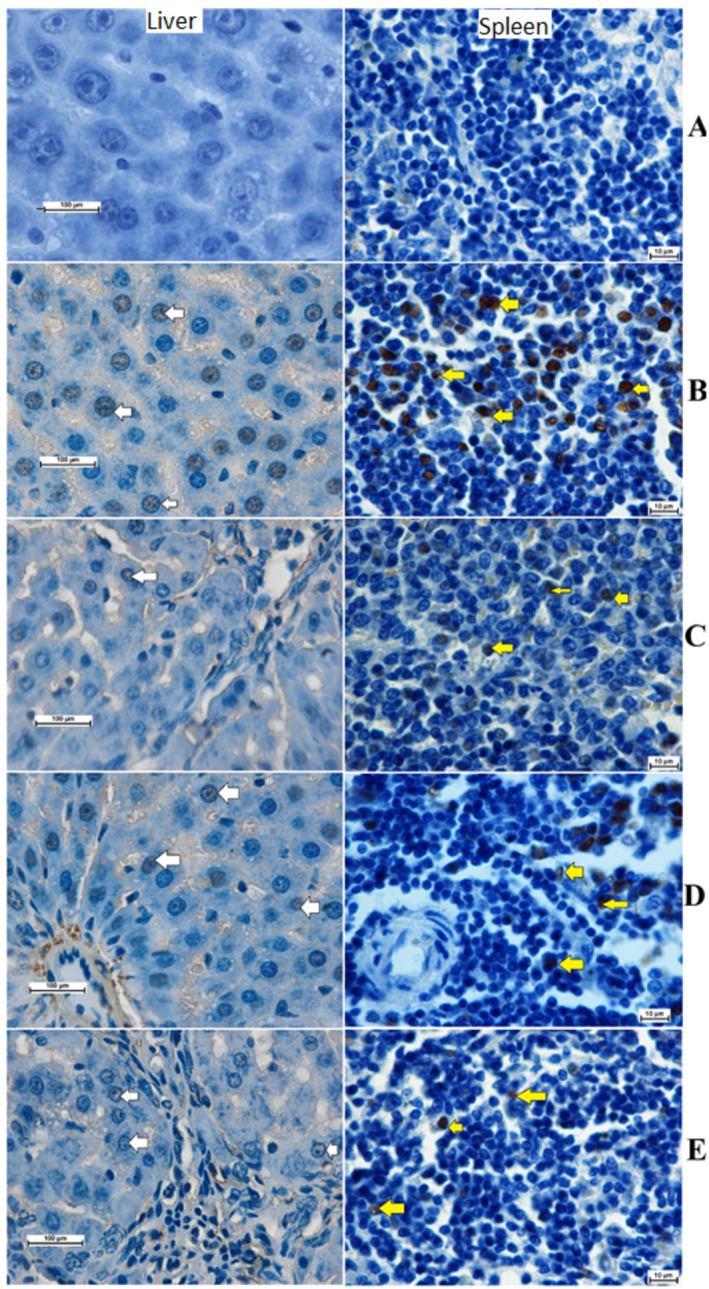
Effect of MEAL on the PCNA expression in liver and spleen tissues. (A) rats received 10% tween 20 + distelled water had no signs of PCNA stains; (B) TAA control rats treated with distal 10% tween 20 + TAA showed severe liver cirrhosis with increased intensity of PCNA stain in liver (white arrow), and increased PCNA in spleen cells (yellow arrow); (C) rats received 50 mg/kg silymarin + TAA revealed Less PCNA‐stained liver cells, indicating hepatic cellular proliferation; (D) rats treated with 250 mg/kg MEAL + TAA had moderate cellular propagation shown by moderate PCNA expression in their liver and spleen cells; (E) rats ingested 500 mg/kg of MEAL + TAA injection showed minor regeneration of liver and spleen cells shown by reduced PCNA expression. Nikon microscope (Y‐THS, Japan). 2× magnification.

The impact of *A. limbata* on cellular proliferation in TAA‐induced hepatotoxicity in rats was evaluated by using immunostaining of proliferating cell nuclear antigen (PCNA) in hepatic and spleen tissues using anti‐PCNA antibody. Normal control rats revealed the absence of any cell renewal in their liver or spleen tissues, represented by the absence of any PCNA expression. The TAA control rats showed increased cell cellular proliferation, mitotic action, and numerous necrotic cells, indicating dissemination to the restoration of extensive liver and spleen tissue damage induced by TAA intoxication. MEAL or silymarin supplementation caused negative modulation of mitotic action, decreased necrotized hepatocytes and spleen cells, and significantly lowered necrotized hepatocytes' proliferation, shown by decreased PCNA staining intensity (Figure 7). These outcomes suggest that one of the hepaprotective actions of MEAL could be through up‐regulation of apoptotic actions in damaged hepatic cells and attenuation effects on the cellular proliferations.

**FIGURE 8:**
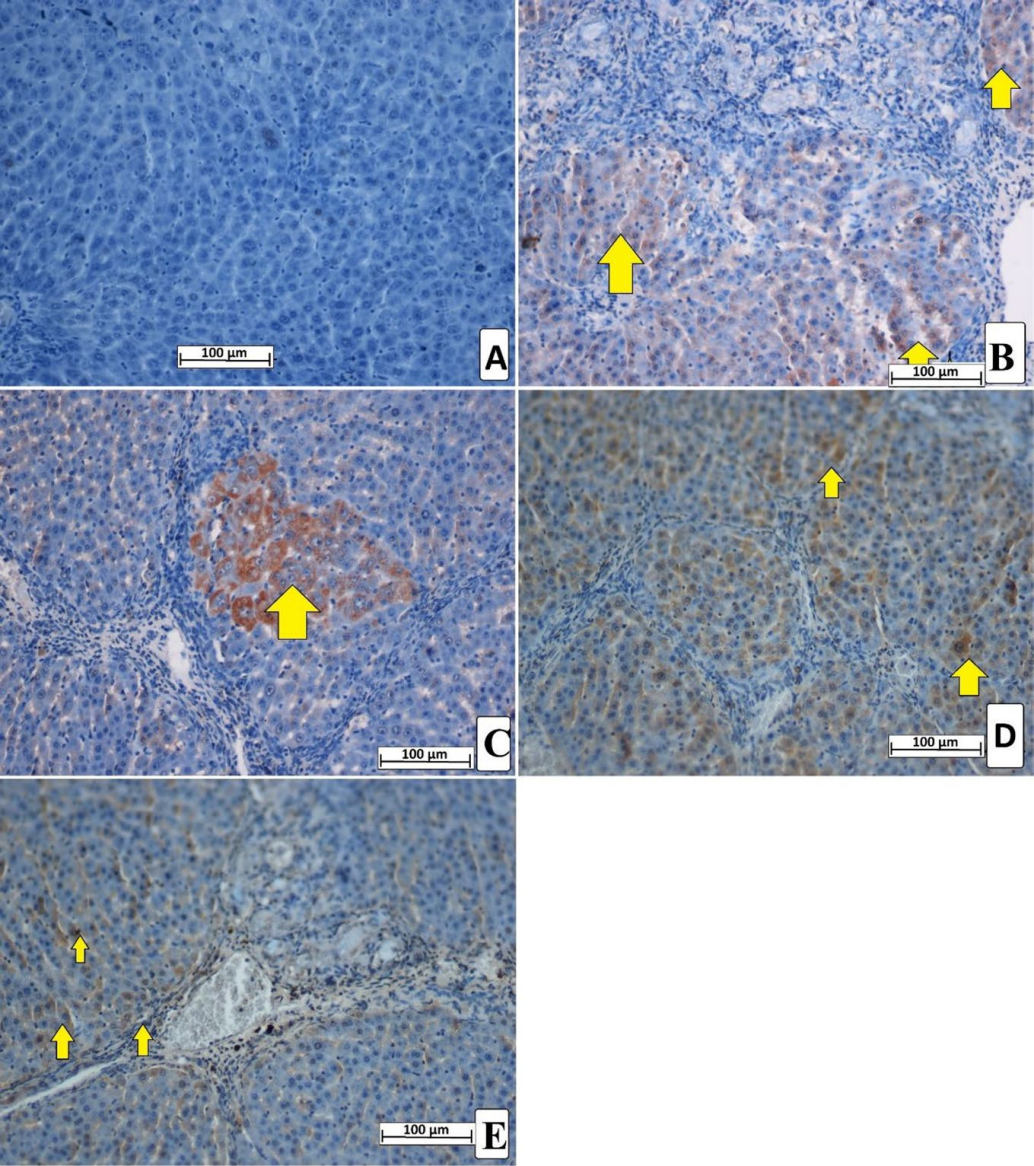
Bax protein expression in different treated rats. (A) normal control rats revealed less apoptosis indicated by reduced Bax positive (yellow arrow) hepatocytes. (B) TAA control rats revealed very reduced Bax proteins denoting lower apoptotic action; (C) silymarine‐treated rats showed moderate expression of moderate Bax staining, indicating moderate apoptosis. (D, E) rats had significantly increased expression of Bax proteins in their liver tissue, indicating large apoptotic actions that eliminate TAA‐injured hepatocytes.

The Bax‐stained hepatocytes from liver tissue obtained from all experimental rats are presented in Figure 8A–E. Hepatocytes from TAA‐control rats showed reduced expression of Bax protein expression, indicating lowered apoptosis and further facilitate cellular proliferation in injured liver tissue (Figure 8B). Silymarin had increased expression of Bax protein, which enhanced apoptotic action in liver tissue (Figure 8C). MEAL‐treated rats (250 and 500 mg/kg) (Figure 8D,E) showed noticeably lower Bax protein appearance in their liver tissue compared to that of TAA controls. The outcomes provide scientific evidence to the idea that MEAL might provoke hepatoprotective actions against sever liver injury by elevating apoptosis of damaged liver cells and slowing their proliferation.

**FIGURE 9:**
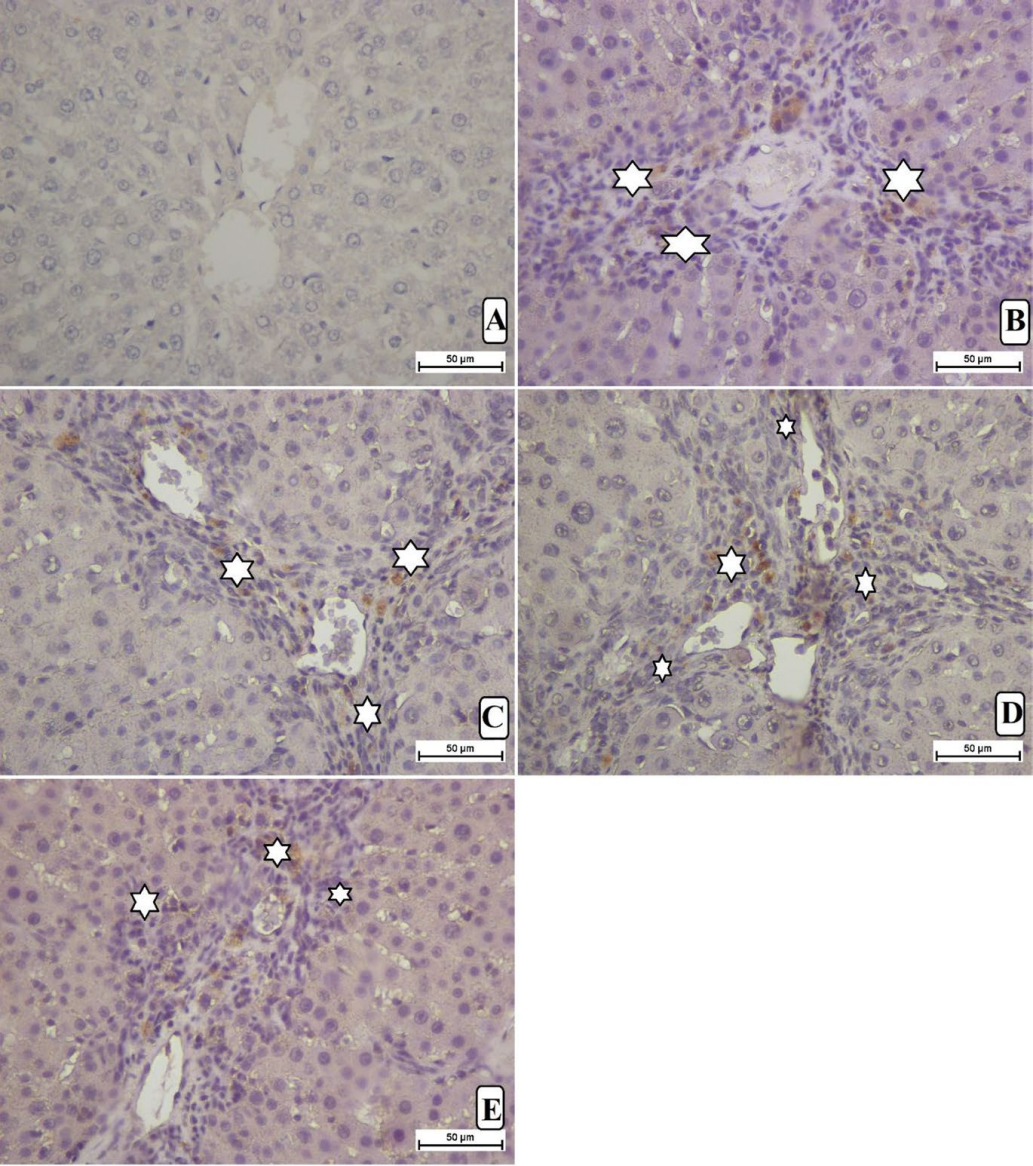
Effect of MEAL supplementation on the TGF‐β1 levels in liver tissue of different treated rats. (A) rats that received 10% tween 20 + distilled water had no signs of TGF‐β1 expression, indicating lack of any cell proliferation and ECM deposition; (B) TAA control rats treated with distal 10% tween 20 + TAA exhibited severe liver cirrhosis with increased cell proliferation (white arrow); (C) rats that received 50 mg/kg silymarin + TAA had reduced the TGF‐β1 expression in their liver tissue; (D) rats treated with 250 mg/kg MEAL + TAA had moderate ECM deposition and hepatocyte proliferation shown by moderate TGF‐β1 expression; (E) rats that ingested 500 mg/kg of MEAL + TAA injection showed minor hepatocyte regeneration presented by significantly less TGF‐β1 expression compared to TAA control rats. Nikon microscope (Y‐THS, Japan). 20× magnification.

The present study revealed different levels of hepatic cell proliferation based on the TGF‐β1 expression in liver tissue. The normal control rats showed the absence of TGF‐β1 intensity in their liver tissue. However, the TAA control rats present intensive expression of TGF‐β1 cytokines, indicating increased inflammatory and immune response, cellular differentiation, increased ECM deposition, proliferation, and apoptotic actions mediated by cytotoxic TAA. Rats treated with silymarin or MEAL (250 and 500 mg/kg) showed significantly less cellular revitalization and mitotic index compared to TAA control rats, denoted by reduced TGF‐β1 appearance in their hepatic tissues. Rats ingested with 500 mg/kg had significant down‐regulation of TGF‐β1 representing lower fibrotic action and reduced cellular propagation (Figure 9).

### 3.4 MEAL Effects on Liver Antioxidants and Oxidative Stress Markers

**FIGURE 10:**
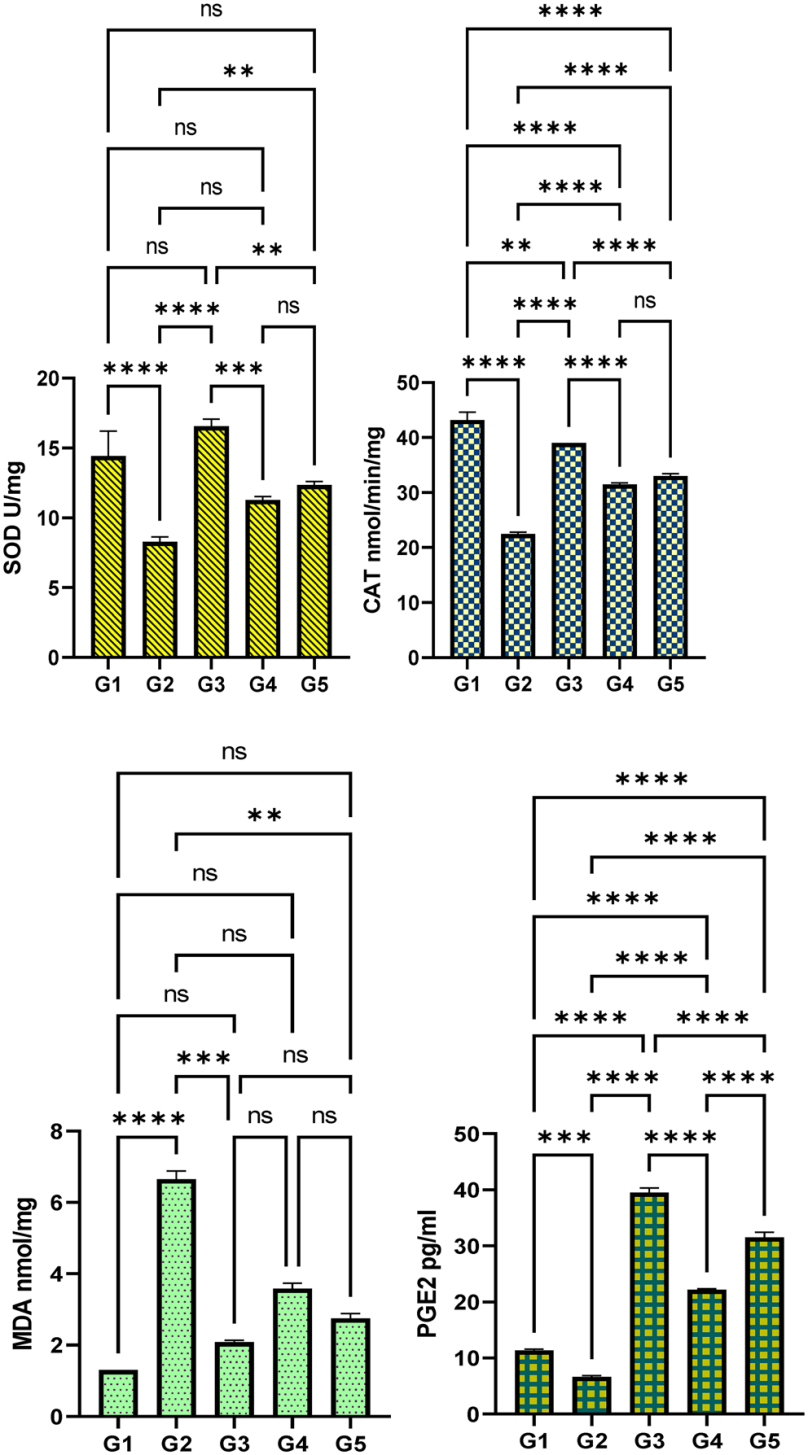
Effect of MEAL on the hepatic antioxidant enzymes and MDA content in TAA‐mediated cirrhosis in rats. (A) rats received 10% tween 20 + distilled water; (B) fibrotic rats treated with 10% tween 20 + TAA; (C) rats received 50 mg/kg silymarin + TAA; (D) rats treated with 250 mg/kg MEAL + TAA; (E) rats ingested 500 mg/kg of MEAL + TAA injection. **, *p* < 0.01; ***, *p* < 0.001; ****, *p* < 0.0001.

The present estimation of the liver antioxidant enzymes and MDA contents are shown in Figure 10. The data analysis showed significant difference in the levels of tissue antioxidants in TAA‐mediated hepatotoxic rats that received treatments. As expected, normal control rats had normal amount of antioxidant enzymes (SOD, 14.43 U/mg; CAT, 43.6 nmol/min/mL; GPx, 11.35) and MDA levels (1.3 U/mg) in their hepatic tissue homogenates. The TAA control rats experienced severe oxidative stress condition, indicated by reduced antioxidant enzymes (SOD, 8.3 U/mg; GPx, 6.63; CAT, 22.58 nmol/min/mL) and elevated MDA (6.65 U/mg) levels in their hepatic homogenates. Silymarine or MEAL (250 and 500 mg/kg) treatment led to suppression of the TAA‐induced oxidative stress in hepatotoxic rats, indicated by increased production of tissue antioxidant and reduced lipid peroxide molecules (MDA) in the hepatic tissues. The silymarine and MEAL‐treated rats showed increased SOD (16.65, 11.26, 12.35 U/mg, respectively), GPX (39.5, 22.16, 31.5 nmol/min/mL, respectively), and CAT (39, 31.56, 32.8 nmol/min/mL, respectively), and lower MDA values (2.08, 3.58, 2.75 U/mg, respectively). The 500 mg/kg MEAL‐treated rats revealed comparable antioxidant enzymes and MDA contents compared to reference group (silymarin‐treated) rats.

### 3.5 Inflammatory Cytokines

**FIGURE 11:**
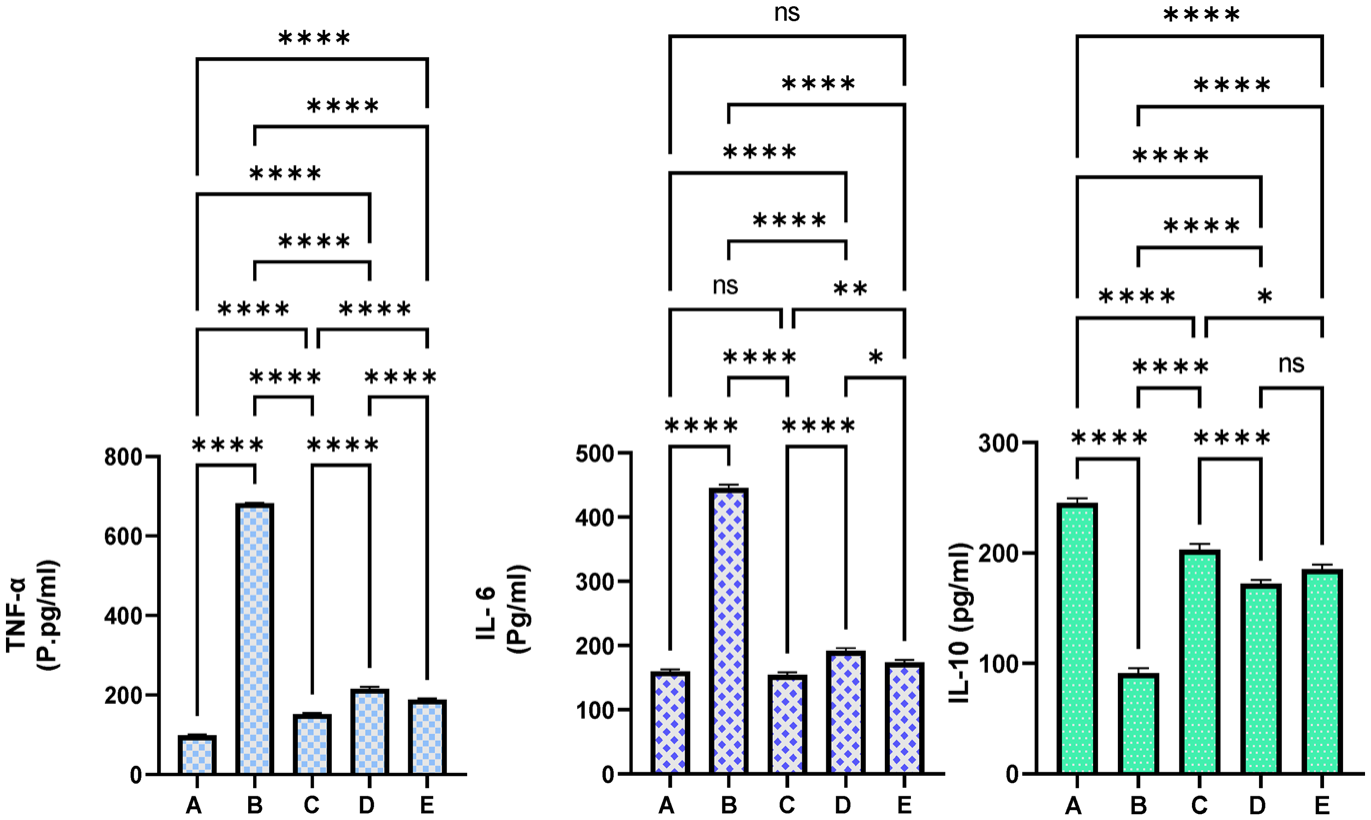
Effect of MEAL on the serum inflammatory markers in rats exposed to hepatotoxicity. (A) rats had 10% tween 20 + distilled water; (B) TAA control rats treated with 10% tween 20 + TAA; (C) rats received 50 mg/kg silymarin + TAA; (D) rats treated with 250 mg/kg MEAL + TAA; and (E) rats ingested 500 mg/kg of MEAL + TAA injection. The TAA induction significantly up‐regulated the serum inflammatory mediators, while silymarin or MEAL treatment resisted TAA‐mediated alterations in the inflammatory mediators, subsequently leading to less liver tissue damages compared to TAA control rats. *, *p* < 0.05; **, *p* < 0.01; ****, *p* < 0.0001.

The serum inflammatory chemicals were significantly modulated as a result of different treatments following TAA injection. As expected, normal control rats showed significantly reduced serum anti‐inflammatory chemicals and higher anti‐inflammatory mediators compared to other experimental rats. The TAA control rats exhibited severe inflammatory conditions, indicated by increased TNF‐α (682.10 pg/mL) and IL‐6 (445.2 pg/mL) and the highest IL‐10 cytokine levels (91.3 pg/mL) than in the other treated groups. The levels of TNF‐α, IL‐6, and IL‐10 were significantly modulated positively in silymarin (152.10, 153.20, and 203.0 pg/mL, respectively) and MEAL 250 mg/kg (188.2, 173.4, and 185.3 pg/mL, respectively) and 500 mg/kg (pg/mL, respectively) MEAL‐treated rats are statistically far away from that of TAA control rats. The present plant supplementation (MEAL) caused significant improvement of serum inflammatory profiles in TAA‐mediated hepatotoxic rats (Figure 11A–E).

### 3.6 Effect of MEAL on Liver Biochemistry

The data results of serum biochemical estimations are shown in Table 4. Normal control rats showed usual liver synthetic and enzymatic production. The obtained biochemical profile indicated that liver injury was indeed provoked in TAA control rats. The TAA control rats exhibit significant enzyme leakage and reduced protein production (albumin) as a result of heavy hepatic injury mediated by TAA. The liver synthetic and excretory function were restored following silymarin or MEAL treatments indicated by higher liver protein (albumin) and lower AST, ALT, bilirubin, and ALP contents in their serum compared to that of TAA control rats (Table 4). Overall, there were non‐significant changes in the serum biochemical between silymarine and MEAL‐supplemented rats, which denotes their hepatprotective effects against TAA‐induced hepatotoxicity to a certain condition near the normalization of the liver synthetic and excretory functions (Table 4).

**TABLE 4:** Effects of MEAL supplementation on the liver biochemical parameters in rats.

Groups	ALP (IU/L)	ALT (IU/L)	AST (IU/L)	Total bilirubin uM	Protein (g/L)	Albumin (g/L)
A	88.37 ± 4.1^a^	62.38 ± 3.4^a^	161.8 ± 3.4^a^	2.23 ± 0.4^a^	64.2 ± 3.1^a^	11.4 ± 1.7^a^
B	229.2 ± 2.9^c^	211.2 ± 4.1^c^	309.2 ± 3.3^c^	7.4 ± 1.1^c^	54.7 ± 2.4^d^	7.8 ± 1.2^c^
C	129.2 ± 3.3^b^	75.2 ± 3.7^b^	179.1 ± 3.7^b^	5.8 ± 0.6^b^	65.2 ± 3.2^b^	12.3 ± 1.5^b^
D	182.8 ± 3.7^b^	85.2 ± 3.2^b^	212.6 ± 3.3^b^	7.7 ± 0.7^b^	58.2 ± 2.4^c^	9.2 ± 1.4^b^
E	149.2 ± 3.2^b^	78.1 ± 3.1^b^	201.4 ± 4.1^b^	5.7 ± 0.9^b^	63.2 ± 3.2^b^	10.3 ± 2.2^b^

*Notes:* Data presented as mean ± SEM; A, rats received 10% tween 200 + distilled water; B, rats treated with 10% tween 20 + TAA; C, rats received 50 mg/kg silymarin + TAA; D, rats treated with 250 mg/kg MEAL + TAA; E, rats ingested 500 mg/kg of MEAL + TAA injection. Values with same letters within column denotes non‐significant at *p* < 0.05.

## 4 Discussion

### 4.1 Phytochemical Content

The present detected polyphenol and flavonoid were found very comparable to previous phytochemical studies of *Anchusa species* (Boskovic et al. 2018; Paun et al. 2020). Similarly, a phytochemical study on *Anchusa undulata L*. reported 130.39 and 122.94 mg/g as polyphenolic contents of roots and aerial parts, respectively, while higher flavonoid contents (117.5 mg/g) were found in aerial parts than that 65.25 g/mg of roots (Zengin and Aktumsek 2015). The discrepancy in the amount of polyphenolic and flavonoid contents from one species to another could be attributed to a number of factors, including changes in the meteorological, petrographic, seasonal harvest, and the growing stage of the plant, along with extraction techniques (Mutha, Tatiya, and Surana 2021).

### 4.2 Acute Toxicity Effect of MEAL

The toxic effects of natural products are considered as early worries that can be paved this way through acute toxicity test, a well‐known indispensable procedure to ensure the safety utilization of any plant with therapeutic interest (Al‐Medhtiy et al. 2024; Jabbar et al. 2024). Previous ethnobotanical studies on *Anchusa* species confirm their safety as herbal therapeutics for various health issues; however, long‐term utilization may lead to some downside effects. The present MEAL supplementation (2 g/kg and 5 g/kg) to rats did not show any toxic damage even after a 14‐day toxicity trial based on the comparable liver and kidney tissue texture and serum biochemical contents compared to normal controls. Currently, systematic search did find any previous records on toxicity evaluation of *Anchusa limbata*. However, oral supplementation with aqueous extract of *Anchusa strigose* did cause any toxic sign or mortality in rats even after 72 h treatment procedure (Muhammed and Arı 2012). Reviewing the available literature established lack of sufficient evidence linked to serious adverse effects following ingestion of *A. limbata* in human. However, future animal and human investigation are required needed to determine its potential downsides.

### 4.3 Hepatoprotective Effect of MEAL

To explore its biological potential and possible molecular mechanisms, we used animal models of liver injury. Intraperitoneal injection of TAA is a well‐known applied method for the investigation of hepatoprotective potentials of natural extracts in animal models, which were similar to that found in humans (Zhang et al. 2019). The present hepatotoxicity procedure revealed significant potential of TAA in provoking liver toxicity in rats after receiving two intraperitoneal injections weekly for 3 weeks. The TAA control rats showed significantly lower body weight and higher liver index compared to other treated rats. Moreover, TAA control rats showed an increased hepatomegaly condition, which could be linked with the increased fat aggregation due to the deterioration of the hepatocytes. This TAA cytotoxicity effect is found similar to the previous studies reporting negative regulation of TAA on the liver weight/body weight ratio in hepatotoxic rats (da Silva et al. 2021; Al‐Attar 2022). In contrast, rats supplemented with silymarin (50 mg/kg) or MEAL (250 mg/kg and 500 mg/kg) had significant restoration of their body weight and liver weight, which were comparable to normal values; this resistant effect of MEAL against TAA might be associated with its present detected phytochemicals (polyphenols and flavonoids). The present hepatoprotective actions of *A. limbata* aerial parts were very comparable with those found by Al‐Snafi et al. (2014), presenting significant hepatoprotective actions of ethanolic and aqueous extracts of *Anchusa strigose* that deactivated the aryl hydrocarbon hydroxylase activity (AHH) and prevented binding of 3H‐benzo (a) pyrene (3H‐BP) to rat liver microsomal protein (Al‐Snafi et al. 2014).

The histopathology of liver tissues obtained from TAA‐control rats revealed intense centrilobular necrosis, congestion of sinusoids, congestion, lobules, subsequently thickened fibrotic septae causing further disruption of the cellular architecture, proliferation of bile duct, dilatation of central veins, and degeneration of liver vacuoles. The obtained histomorphology characteristics were comparable with those found in the previous studies evaluated for thioacetamide control rats (Guo et al. 2024). However, silymarin or MEAL‐treated rats exhibited mild necrosis, less necrosis, and restored those liver tissue changes mediated by TAA hepatotoxicity. Histopathological evaluation using Masson's trichrome technique revealed increased collagen deposition around the central veins of TAA control rats, indicating a noticeable alteration of the membrane permeability of hepatocytes. The silymarin or MEAL (250 mg/kg and 500 mg/kg)‐treated rats showed noticeable liver recovery from TAA hepatotoxicity, and the hepatic architecture was noticeably less collagen deposition compared to TAA control rats. Accordingly, researchers showed significant modulatory potentials of aerial part extracts of *Anchusa* species on the collagen deposition in the excisional wound model, which were mainly linked with its phytochemical contents (phenolics and flavonoids and saponins) (Al‐Qaisi et al. 2024).

### 4.4 Effect of MEAL on Immunohistochemicals (Bax and PCNA)

PCNA immunoreactivity is a widely applied technique for the evaluation of cellular proliferation in normal, proliferative, and cancerous livers in rat models. Cell proliferation is an important step in any cellular regeneration response toward injurious agents and is considered a pivotal factor for cancer initiation (Abood et al. 2020). In the current tissue evaluation, PCNA protein expression was noticeable at its highest in the hepatic tissues of TAA control rats, indicating an increasing rate of cirrhosis, cellular proliferation, and reduced tissue regenerations. However, silymarin or MEAL (250 mg/kg and 500 mg/kg)‐treated rats revealed less PCNA expression in their liver tissue, denoting fewer fibroblast production and cellular proliferation.

Bax protein is a well‐known pro‐apoptotic protein that is considered a core modulator of the intrinsic mechanism of apoptosis. They become activated during apoptotic initiation, and they become oligomerized at the outer membrane of mitochondria to facilitate its release, an important step in apoptosis. In the present study, TAA control rats revealed reduced expression, indicating lowered apoptosis and further facilitating cellular proliferation in injured liver tissue. MEAL treatment caused significant up‐regulation of Bax protein in their hepatic tissues, facilitating higher apoptotic actions for damaged hepatocytes. Such immunomodulatory actions of MEAL could be attributed to its phytochemical profiles (polyphenols and flavonoids). Accordingly, numerous reports declared the immunomodulatory effects of *Anchusa* species and its related chemical compounds (Wang et al. 2020; Mahmood, Abd, and Qasim 2021). Similarly, the extraction and its isolated compounds from aerial parts of *Anchusa italica* have shown significant modulatory effects on the anti‐apoptotic Bcl‐2 and pro‐apoptotic Bax protein expression in hypoxia/reoxygenation (H/R) induced cardiomyocytes injury in neonatal rats (Hu et al. 2020).

### 4.5 Effect of MEAL on TGF‐β1

The initiation and progression of liver fibrosis and cirrhosis include several mechanisms that are mediated by inflammatory mediators, growth factors, and immunomodulators such as transforming growth factor‐β (TGF‐β1). TGF‐β1 is considered as the most important pro‐fibrogenic cytokine in fibrosis initiation and numerous physiological pathways; therefore, understanding molecular pathways associated with TGF‐β1 is an essential step toward therapeutic inventions. TGF‐β1 can alter the accumulation of the extracellular matrix by different mechanisms based on the severity of liver injuries (mainly hepatic stellate cells). After acute liver damage mediated by chemicals (TAA), TGF‐β1 secretion provokes collagen synthesis by provoking hepatic stellate cells by the Smad pathways such as in a fibrotic rat model. In the present tissue examination, the TAA control rats exhibited significantly highest TGF‐β1 expression, which is similar to those findings that evidenced the positive modulation effect of TAA in liver tissue in different rat trials (Ramos‐Tovar et al. 2018; El‐Baz, Salama, and Salama 2019). However, silyamarin or MEAL (250 mg/kg and 500 mg/kg)‐treated rats showed significantly less TGF‐ β1 suppression and fewer histological fibrosis compared to TAA controls. The inhibitory potentials of MEAL inhibitory actions against TGF‐ β1 could be linked with its detected chemical profiles (polyphenols and flavonoids), which were repeatedly shown as effective inhibitors of this pro‐fibrogenic cytokine (Yan et al. 2020; Xu et al. 2022; Wan et al. 2023).

### 4.6 Effect of MEAL on Oxidative Stress

Oxidative stress plays an important role in numerous liver diseases and is a crucial risk factor for the progression of liver injury. The tissue antioxidants (SOD, CAT, and GPx) are well‐known biomarkers for the estimation of oxidative stress rate and redox state (Ullah et al. 2023). In the present data, TAA control rats experienced the highest oxidative stress represented by reduced tissue antioxidants (SOD, CAT, and GPx) and increased amount of endogenous MDA contents in the hepatic tissue subsequently provoking various inflammatory pathways and further liver tissue damage, as researchers declared (Yang, Cho, and Hwang 2022). Accordingly, researchers have shown TAA efficacy in the negative modulation of antioxidants and positive regulation of ROS molecules in liver tissues, which could be one of the mechanisms by which TAA leads to tissue necrosis and liver cirrhosis (Jabbar et al. 2023a). Silymarin or MEAL treatment caused significant up‐regulation of tissue antioxidants and down‐regulation of lipid peroxidation (MAD levels), denoting lower oxidative tissue damage which might be coordinated with its mitigation effect of TAA‐mediated hepatotoxicity. Similarly, the antioxidant potentials of *Anchusa* species, *A. italica* (Khomsi et al. 2022), *A. arvensis* (Hussain et al. 2023), *A. Ovata* (Jaradat et al. 2020), and *A. azurea* (Kiziltaş 2022) reported previously were mainly linked with its phytochemical contents (Polyphenols and flavonoids).

### 4.7 Effect of MEAL on Inflammation

The inflammatory mediators can have provoking action on the gradual scaring damage of hepatic tissues subsequently causing liver cirrhosis. The tumor necrosis factor (TNF‐α), interleukin‐1 β (IL‐1 β), and interleukin‐6 are pro‐inflammatory cytokines that can enhance the progression of cirrhosis through modulation of various cellular processes including lipid metabolism, biliary system obstruction, positive and negative acute phase proteins, and cirrhosis progression (Slautin et al. 2023). The production of many inflammatory chemicals is majorly modulated by the transcription nuclear factor‐kappa B (NF‐_k_B). Moreover, the NF‐κB pathway can modulate inflammation, cell differentiation or proliferation, and oxidative stress‐related disorders. NF‐κB pathway can be provoked by a wide diverse factor and a complex network of cellular mechanisms including inflammation cascade, thus forming a cycle of auto‐regulation of the inflammatory response for a prolonged time (Hangda et al. 2024). In the present study, TAA control rats showed increased serum inflammatory cytokines and reduced anti‐inflammatory cytokines compared to all experimental rats. However, silymarin or MEAL supplementation restored TAA‐mediated inflammation, shown by lower TNF‐α and IL‐6 and higher IL‐10 cytokines compared to fibrotic rats. Similarly, researchers reported significant anti‐inflammatory and immunomodulatory activities of ethanolic extracts from *Anchusa officinalis* in an in vitro trial, which were mainly linked with its polyphenolic (rosmarinic acid) and flavonoid (luteolin) (Paun et al. 2020). The hydroalcoholic extracts of aerial parts of *A. italica* exhibited significant anti‐inflammatory potentials shown by down‐regulation of TNF‐α, IL‐1β, and IL‐6 in myocardial infarction mice model, which were mainly linked with its total flavonoid and polyphenolic contents (Wang et al. 2020).

### 4.8 Effect of MEAL on Liver Enzymes and Protein

The serum biochemical profiling including estimation of liver enzymes (ALP, ALT, AST, GGT), waste product (bilirubin), and liver proteins (total protein, and albumin) is considered a reliable procedure for evaluating liver functionality and liver toxicity. The leakage of liver enzymes into the circulated blood can give clear indications of the severity level of the liver injury. Moreover, a significant drop in liver proteins may result from malnutrition or liver tissue injury (Sun et al. 2023). In the present experiment, TAA inoculation caused a noticeable elevation of the liver enzymes and decreased serum proteins, indicating increased liver dysfunctionality. Accordingly, scientists have correlated the TAA stimulatory effects of liver enzymes with its interaction with genetic materials (DNA and RNA), which further injured hepatic tissues (Miao et al. 2020). The TAA administration also down‐regulated the total protein and albumin levels, which were mostly linked with its inhibitory action on the transcriptional pathways (mRNA) and enhancement of the nuclei acid leakage from the nucleus to the cytoplasm (Ghanim et al. 2022). The present MEAL supplementation resisted TAA‐hepatotoxicity and restored liver biomarkers parallel to that of silymarin. Accordingly, ethanolic extracts (50 mg/kg, 100 mg/kg, and 200 mg/kg) of *A. italica* improved serum biochemical profiles in Pentylenetetrazole‐Induced Seizure in Mice, which were mainly linked with its total flavonoid and polyphenolic contents (Rahimi‐Madiseh et al. 2021).

## 5 Conclusions

The safety evaluation of MEAL revealed a lack of any toxic damage in rats ingested with up to 5 g/kg, which was supported by biochemical and histology indicators. Evidenced by its increased polyphenols and flavonoid contents, *MEAL* showed significant hepatoprotective potentials against TAA‐induced hepatotoxicity in rats. The hepatoprotective action of *MEAL* could be linked to its phytochemical potential to down‐regulate hepatocyte proliferation (mitotic action), reduce collagen deposition, and strengthen the liver defense mechanism. MEAL supplementation improved immunohistochemical expressions (decreased PCNA and increased pro‐apoptotic Bax protein) as well as decreased TGF‐β1 expression in liver tissues. Moreover, MEAL treatment resisted TAA‐stress oxidative stress in hepatotoxic rats, indicated by increased SOD, CAT, and PGE2 enzymes and reduced MDA contents. MEAL‐treated rats exhibited significant anti‐inflammatory potentials against TAA‐mediated hepatotoxicity shown by lower TNF‐α and IL‐6 and higher IL‐10 levels compared to the cirrhotic positive model. The current study limitations (small sample size, poor facility, and lack of laboratory equipment) encourage scientists to conduct further experiments on the molecular characterization of *A. limbata* responsible for its biological potential.

## Author Contributions

**Khaled Abdul‐Aziz Ahmed:** resources (equal), software (equal), supervision (equal), validation (equal). **Ahmed A. j. Jabbar:** conceptualization, Writing original draft. **Mohammed M. Hussein M. Raouf:** resources (equal), software (equal), supervision (equal), validation (equal). **Ayman M. Al‐Qaaneh:** resources (equal), software (equal), supervision (equal), validation (equal). **Rawaz Rizgar Hassan:** resources (equal), software (equal), supervision (equal). **Musher Ismael Salih:** formal analysis (equal), software (equal), supervision (equal), validation (equal). **Ramzi A. Mothana:** Funding. **Gadah Abdulaziz Al‐Hamoud:** formal analysis (equal), software (equal), supervision (equal), validation (equal). **Mahmood Ameen Abdulla:** Conceptualization. **Sidgi Hasson:** resources (equal), software (equal), supervision (equal), validation (equal). **Sidgi Hasson:** resources (equal), software (equal), supervision (equal), validation (equal). **Parween Abdulsamad Ismail:** data curation (equal), formal analysis (equal), software (equal), validation (equal).

## Consent

The authors have nothing to report.

## Conflicts of Interest

The authors declare no conflicts of interest.

## Data Availability

Data regarding the current study are provided within data repository.
