# Correction to “Phytochemical Profiling, Acute Toxicity, and Hepatoprotective Effects of Anchusa Limbata in Thioacetamide‐Induced Liver Cirrhosis in Rats”

**DOI:** 10.1002/fsn3.70255

**Published:** 2025-05-13

K. Abdul‐Aziz Ahmed, A. A. J. Jabbar, M. M. Hussein M. Raouf, A. M. Al‐Qaaneh, R. R. Hassan, M. I. Salih, R. A. Mothana, G. A. Al‐Hamoud, M. A. Abdulla, S. Hasson and P. Abdul‐samad Ismail, “Phytochemical Profiling, Acute Toxicity, and Hepatoprotective Effects of Anchusa Limbata in Thioacetamide‐Induced Liver Cirrhosis in Rats,” *Food Science and Nutrition* 12, no. 12 (2024): 10628–10645, https://doi.org/10.1002/fsn3.4544.

Following publication of this article, it was noted by a third party that the liver image shown in Figure 4C contained an overlap with another image published by the same authors elsewhere. The authors acknowledge the overlap and explained that it was an inadvertent error due to figure mismanagement. The authors have corrected Figure 4 by supplying alternative images for the entire figure to maintain consistent H&E intensity. The authors confirm that all the experimental results and corresponding conclusions mentioned in the paper remain unaffected and sincerely apologize for the mistake.

**Figure:**
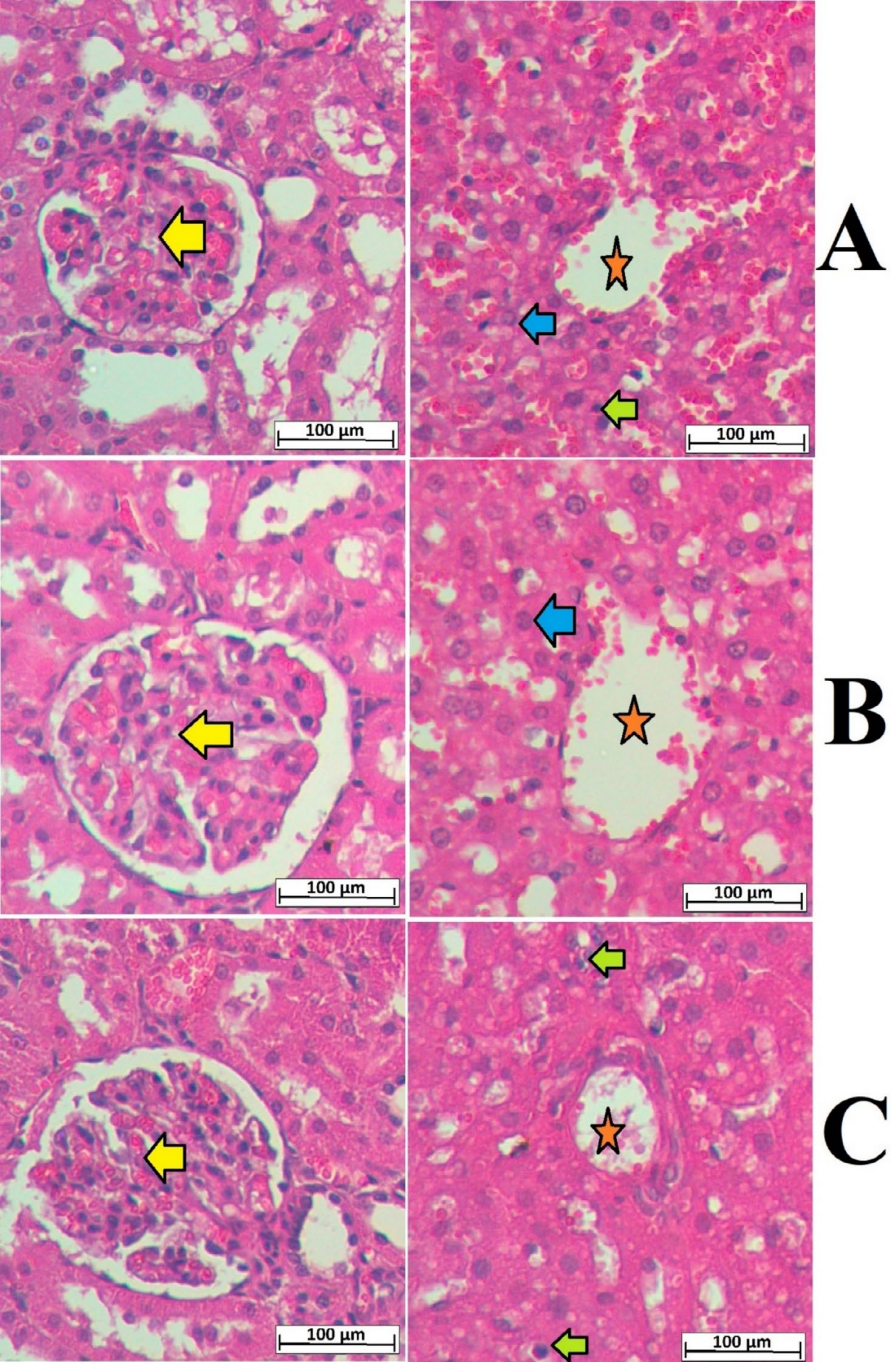
**FIGURE 4** Microscopic presentation of the liver and kidney tissues of rats in acute toxicity trial. (A) normal controls fed on 10% tween 20; (B) rats ingested 2 g/kg MEAL; (C) rats ingested 5 g/kg MEAL. The alignment of the kidney and liver histological layers was very comparable between normal control and MEAL‐treated rats. The kidney tissue appeared as a normal bowman's capsule with glomeruli (yellow arrow) and adequate interlobular blood vessels as well as distal convoluted tubule and proximal convoluted tubules. The hepatic tissues appeared with a central vein (orange asterisk); Kupffer cell (green arrow), and normal liver cell with circular nucleus (blue arrow) for all tested rats (hematoxylin and eosin stain, magnification 20×).

